# Bullous pemphigoid associated with the use of dipeptidil peptidase-4 inhibitors: analysis from studies based on pharmacovigilance databases

**DOI:** 10.1007/s11096-020-01003-6

**Published:** 2020-03-05

**Authors:** Juan A. Molina-Guarneros, María Sainz-Gil, Rosario Sanz-Fadrique, Pilar García, Pedro Rodríguez-Jiménez, Ester Navarro-García, Luis H. Martin

**Affiliations:** 1 Department of Pharmacology, Faculty of Medicine, National Autonomous University of Mexico, Mexico City, Mexico; 2 Centre for Drug Safety (CESME), Faculty of Medicine, Valladolid University, Valladolid, Spain; 3 Centre for Pharmacovigilance of Castilla y León, Valladolid, Spain; 4 Primary Healthcare Centre ‘Covaresa’, Valladolid, Spain; 5 “La Princesa” Hospital, Madrid, Spain; 6 University Hospital ‘Río Hortega’, Valladolid, Spain

**Keywords:** Bullous pemphigoid, Case-non case study, Dipeptidase-4 inhibitors, Gliptins, Pharmacovigilance

## Abstract

*Background* Bullous pemphigoid has been associated to dipeptidase-4 inhibitors. *Objectives* Addressing the potential Bullous pemphigoid-dipeptidase-4 inhibitors association based on pharmacovigilance data currently available in Spain in order to obtain a composite disproportionality estimator from all the data generated by the case-non case studies conducted to this date. *Setting* The Spanish Pharmacovigilance System for Human Use Drugs database. *Method* Case-non case study based on the Spanish Pharmacovigilance System for Human Use Drugs notifications submitted between 2007 and 2018 (n = 169,280), using the Medical Dictionary for Regulatory Activities term (Preferred Term) ‘pemphigoid’ for sitagliptin, vildagliptin, saxagliptin, linagliptin, and alogliptin (n = 1952). As negative control, we used acetaminophen, while furosemide was the positive control. A pooled reported odds ratio analysis in the French, Japanese, and Spanish national pharmacovigilance databases was performed. On The Spanish Pharmacovigilance System for Human Use Drugs, we conducted a bullous pemphigoid-metformin association analysis within the period 1982–2018. *Main outcome measure* Adverse reaction cases in pharmacovigilance databases and the disproportionality through the reporting odds ratio. *Results* Within The Spanish Pharmacovigilance System for Human Use Drugs, we found 45 cases of bullous pemphigoid in dipeptidase-4 inhibitors patients. Median age was 77 years (range 72–82). The median latency period was 7 months (range 0.23–86). The Bullous pemphigoid-dipeptidase-4 inhibitors association was established with a reporting odd ratio = 70.0 (95% confidence intervals 49.1–10.1). In the combined analysis of the three aforementioned pharmacovigilance databases, the pooled reporting odd ratio was 81.0 (95% confidence intervals 69.5–94.4). *Conclusion* The composite estimator for the three national pharmacovigilance databases yields clear evidence of a Bullous pemphigoid-dipeptidase-4 inhibitors association, which was statistically significant for both the pharmacological class as a whole and each of the dipeptidase-4 inhibitors agents under investigation. Metformin’s role in the incidence of bullous pemphigoid appeared casual rather than causal. No differences between Caucasian and Asian populations were noted.

## Impacts on practice

- Patients undergoing gliptin treatment should also be seen by a dermatologist, to check for possible Bullous pemphigoid.
- In the future, it could be helpful to establish risk groups for Bullous pemphigoid, accounting for exposition period, age, and gender.

## Introduction

In Spain, the first approved gliptin was sitagliptin (March 2007), followed by vildagliptin (October 2007), saxagliptin (November 2009), linagliptin (September 2011), and alogliptin (January 2014). Gliptins are used in monotherapy [1]. As per American Diabetes Association (ADA) and European Association for the Study of Diabetes (EASD) recommendations, metformin is marked as the first-line choice where not contraindicated. Dipeptidil peptidase-4 inhibitors (DPP-4-i) can be used as monotherapy when metformin is contraindicated or not tolerated. Some studies have shown the value of initial metformin-DPP-4 inhibitor combination therapy in special populations. However, most often, they are prescribed in combination with metformin [2]. In Spain, metformin is the first pharmacological choice to treat DM2, whereas i-DPP4 are the first indication on serious renal insufficiency patients (glomerular filtration under 30 ml/min, FG < 30 ml/min) and the second choice for patients over 75 years old [3]. I-DDP4s stimulate insulin secretion. They inhibit the enzyme dipeptidyl-peptidase 4 (DPP-4) whose function is GLP-1 (glucagon-like peptide hydrolyzation). When food goes into the intestine, this peptide is released therein, which results in an increase of insulin release and the inhibition of glucagon, in a glucose-dependent process.

In the post-commercialisation period, iDPP4s have been associated to several hypersensibility ADRs which may become life-threatening, such as hives, angioedema, and pancreatitis (Saxagliptin). Gliptins have also been recently associated with a skin condition called bullous pemphigoid (BP) [4], and their labels now specify this adverse reaction [1, 2]. BP is a serious pathology requiring systematic and continuous treatment. Its evolution is chronic and variable. Even though it usually concludes within a period of 5 years, there is a moderate mortality rate associated to this disease and its treatment. Since its aggressiveness and treatment response may widely vary, there are no precise studies on the effect that BP may have on patients’ lives.

Recently, some studies based on pharmacovigilance databases showed an association between gliptins and BP [5, 6]. The association became first apparent through the publication of cases and case series reporting concrete patients, but more knowledge has developped through disproportionality analysis, which allows estimators (PRR, ROR, X^2^) yielding information on the association level between iDPP4 and BP. There are two reports on French and Japanese [5, 6] pharmacovigilance databases, hence our study has focused on statistical estimators within the Spanish database.

This association was further strengthened by actual data on a probable incidence. Two retrospective analyses demonstrated that DPP4i are associated with an increase of BP risk, with adjusted ORs of 67.5 (95% CI 47.1–96.9) [7] and 2.64 (95% CI 1.19– 5.85) [8].

Aiming to improve the knowledge on this potential association, in 2018 we conducted a case/non-case study based on the Spanish Pharmacovigilance System of Human Medicines (SEFV-H, according to its Spanish acronym) database (FEDRA). Up to date, the established fact, apparently confirmed in our study, is a statistically significant association which will not allow to taxatively deduce any cause-effect relationship. We may thus speak about an increase of BP risk after the intake of iDPP4, just as estimators (mainly OR) obtained in observational studies indicate. We can also confirm an increase on the possibility of pharmacovigilance reports of BP among iDPP4 patients, although these estimators, which draw a statistic notion of the association’s strength, do not yield a cause-effect bond yet.

The potential association between BP and some antidiabetic drugs, like sitagliptin and vildagliptin [4], has been linked to BP triggering antigens, such as antigen 1 (BP230), a component of the hemidesmosomal plaque, and antigen 2 (BP180), a transmembrane protein. These antigens are components of hemidesmosomes located in the lucid lamina of the basal membrane. They are proteins of 230 kDa and 180 kDa, respectively. Most BP patients present autoantibodies which bind to the 180-kDa protein immunodominant region, referred to as the non-collagenous domain NC16A [9, 10]. However, in patients with gliptin-induced BP, most of these autoantibodies have been reported to react to other sites of the BP180 protein, such as LAD-1 and/or the terminal carboxyl domain [11]. To our knowledge, no specific BP drug-inducing autoantibodies have been identified yet. Such antibodies are speculated to be the same as those generated by the spontaneous inflammatory or non-inflammatory BP. In BP patients, Giusti [12] found a predictive biomarker for the eosinophil cationic protein (ECP) remission in serum concentration. More recently, the differential effects between IL17-A and IL-23 in BP patients has been demonstrated in ex vivo experiments [13].

SEFV-H database contains 277,351 spontaneous notifications of suspected adverse drug reactions (ADRs). Since two studies on this drug-ADR coupling have been previously published based on the national French [5] and Japanese [6] databases in 2016 and 2018, respectively, we decided to also conduct an analysis by combining the data from the French and Japanese studies with our own. Additionally, we attempted to determine whether this potential association is affected by either the geographical distribution of a particular ethnicity or the prescribed IDPP4 inhibitor agent. Likewise, we addressed the potential link between the BP-IDPP4 association and patients’ demographic variables, such as age and sex. On the other hand, in clinical practice, it is usual to co-administer gliptins and metformin, which may constitute a double confounding factor in analysing the data in our study. In Spain, metformin commercialisation was approved in 1982. Despite BP not being labeled as a potential ADR to metformin [14], there is a reasonable concern about metformin’s involvement with this skin condition; therefore, we also addressed the potential association between metformin and BP.

## Aim of the study

Addressing the potential BP-IDPP4 association based on pharmacovigilance data currently available in Spain in order to obtain a composite disproportionality estimator from all the data generated by the case-non case studies conducted to this date. Also, to assess the possible role of metformin in the occurrence of this adverse reaction.

## Ethics approval

Since the study consisted in revising pharmacovigilance databases without patient contact, no approval is required.

## Methods

We implemented a case/non-case study based on the SEFV-H database (FEDRA). Health professionals, citizens and the pharmaceutical industry reports cases to SEFV-H. Reporting is mandatory for professionals and industry. Reports may come from studies or be spontaneously notified. Spontaneous reports can either be sent directly to SEFV-H, preferably, or through the pharmaceutical industry. Cases published in the literature enter FEDRA as well. FEDRA contains data from 1982 to 30th November 2018, including 277,351 spontaneously notified cases of suspected ADRs. The case/non-case approach is used to estimate the disproportionality measure between the expected and reported cases for either a given drug or a pharmacological class as a whole, on the one hand, and a specific ADR, on the other, according to the data entered in a specific database [15]. The notifications including the ADR of interest are considered ‘cases’, while the remainder are considered ‘non-case’. To search the active ingredients (i.e. sitaglipin, vildagliptin, saxagliptin, linagliptin, and alogliptin) and the cases within 26-March-2007/30-November-2018, the MedDRA V21.0 preferred term (PT) (i.e. “Pemphigoid”) was used. As a negative control (it is known not to cause BP) and a positive control (it is known to cause BP), we selected acetaminophen and furosemide, respectively [5, 6]. Since the dictionary in use prior to 2007 (WHO-ART) did not include the term “Pemphigoid”, for the BP-metformin association analysis, the research before 2007 was implemented under the term “pemphigus”.

### Data statistical analysis

In the absence of disproportionality, it is assumed that exposure to the drug under investigation and ADR occurrence constitute independent phenomena. The disproportionality in a given relationship between the drug and the ADR found in a pharmacovigilance database may be indicative of the occurrence of a drug-ADR association. A 2X2 contingency table based on a given database is used to estimate the disproportionality indicator (Table 1).

**Table 1:** 2 × 2 contingency table

Drug of interest	Adverse reaction of interest	
	cases	non cases
Exposed	*a*	*b*
Non exposed	*c*	*d*

To estimate the disproportionality, we used the reporting odds ratio (ROR) [16]. To estimate the ROR along with its 95% CIs and χ^2^, we used the Epi Info V 7.2.2.6. (24/01/2018) program. The ROR (95% CI) was estimated for the set of IDPP4 agents as a whole, as well as for each gliptin individually and by gender. Furthermore, we conducted a sensitivity analysis. To this purpose, those cases including other medications (with the exception of gliptins) whose association with BP is well established were removed from the analysis. For this, a clinical dermatologist (PJ), a primary healthcare physician (PO), and a pharmacologist (JM) reviewed separately every notification individually. The cases including any other medication (either co-administered or suspected) associated with BP in the medical literature were discarded [4].

### Pooled analysis from the studies conducted over the three national databases

A search in PubMed with the terms “DPP4 inhibitors”, “bullous pemphigoid” and “case non-case studies” was performed. As a result, we found a paper on disproportionality analysis based on the national Japanese database of adverse drug reaction notifications [6]. A repeated search into the Cochrane database with the terms “bullous pemphigoid” and “DPP4i” yielded negative results. Lastly, we gathered the data published in the medical literature by Bene [5] based on the national French Pharmacovigilance System Database (FPVD), as well as the data published by Arai based in the national Japanese Pharmacovigilance Database (JADER) [6]. Once the total number of cases/non-cases was obtained, the pooled disproportionality was estimated (i.e. ROR from the three national databases). Forest plot graphics were generated by means of the software Review Manager (RevMan) Version 5.3.

## Results

Within the study period, FEDRA enclosed 169,180 cases of suspected ADRs. Out of these cases, 1998 included a DDP4i agent, and in 45 (2.3%) of them, the reported ADR was BP. In 44 of these cases, gliptin was considered the suspected medication; only in 1 case, was the concomitant drug the suspected cause (in this case, the temporal sequence was unreported). Gliptins were reported as follows across these ADRs: vildagliptin, 28; linagliptin, 10; sitagliptin, 6; saxagliptin, 1. There were no notifications involving alogliptin. The affected patients’ (n = 43) median age was 77 years (range 72–82). There were no statistically significant differences between males and females (P = 0.60). Between the start of gliptin therapy and the first BP occurrence, the median latency was 7 months (range 0.23–86 months). Regarding active ingredients, median latency for vildagliptin was 11 months (n = 23), 3.5 months for linagliptin (n = 10), and 12 months (n = 3) for sitagliptin. In 23 cases, the patients were reported to receive ADR treatment (corticoids in all cases). In 21 cases, this information was unreported. In 1 case, the patient was re-exposed to gliptin. This was an 81-year-old male who presented BP following 12-months of therapy with co-administered metformin and vildagliptin. Two months after diagnosing BP, gliptin was discontinued and he was treated with oral corticoids. The clinical picture subsided then; however, BP remission was not complete. Three months later, the patient was re-exposed to vildagliptin, and the lesions exacerbated. Again, he was treated with oral corticoids (prednisone), but he continued on vildagliptin for 8 additional months after which the gliptin was definitely suspended and the final clinical outcome was unreported. On the other hand, 24 patients received only one gliptin as an antidiabetic medication—11 vildagliptin, 9 linagliptin, 3 sitagliptin, and 1 saxagliptin–. In addition, 21 patients were treated with concomitant metformin—17 on vildagliptin, 3 on sitagliptin, and 1 on linagliptin.

### Disproportionality analysis

Disproportionality analysis based on FEDRA data showed an association between gliptins and BP, with ROR (95% CI) being 70.0 (47.1–104.1) (Table 2). The largest disproportionality corresponded to vildagliptin, followed in decreasing order by linagliptin, saxagliptin, and sitagliptin. The association differences between the individual gliptins yielded statistically significant results only between sitagliptin, on the one hand, and the remaining gliptins, on the other. For acetaminophen and furosemide, RORs (95% CI) were 0.4 (0.1–1.5) and 5.5 (2.9–10.7), respectively.

**Table 2:** Association between DPP4i exposure and bullous pemphigoid reports as measured by the disproportionality analysis based on FEDRA database

Active ingredient	Cases/non-cases	ROR (95% CI)	X^2^
*All drugs*^*a*^	*99/169,180*	**–**	**–**
*All DPP4i*	*45/1953*	*71.4 (47.9*–*106.3)*	*1609.8*
Females	22/964	91.2 (50.9–163.2)	*955.8*
Males	23/971	52. 2 (30.2–90.2)	*611.0*
*Vildagliptin*	*29/601*	*113.9 (73.4*–*177,0)*	*2115.4*
Females	*15/278*	*170.3 (90.6*–*3200)*	*491.5*
Males	*14/318*	*83.4 (45.3*–*153.5)*	*695.8*
*Linagliptin*	*10/300*	*55.2 (28.2*–*108.0)*	*408.3*
Females	*6/150*	*88.2 (36.2*–*214.5)*	*349.3*
Males	*4/148*	*31.6 (11.1*–*89.3)*	*79.6*
*Sitagliptin*	*5/981*	*9.1 (3.7*–*22.6)*	*27.1*
Females	*1/490*	*4.4 (0.6*–*32.0)*	*0.3*
Males	*4/479*	*11.3 (4.0*–*31.5)*	*25.6*
*Saxagliptin*	*1/58*	*27.4 (3.7*–*200.1)*	*5.71*
Females	0*/36*	N.C.^**b**^	N.C.^**b**^
Males	1/*22*	54.4 (7.1–411.8)	11.7
*Furosemide*	*10/3329*	*5.5 (2.9*–*10.7)*	26.0
*Acetaminophen*	*2/8788*	*0.4 (0.1*–*1.5)*	1.4

^a^From first DDP4i approval date

^b^Not calculable

### Sensitivity analysis

In 14 cases, the patients were being or had been treated with other medications, for which BP has been established as an ADR [4]; namely: furosemide, 6 cases; omeprazole, 6; amlodipine, 4; spironolactone, 1; and enalapril, 2. To determine whether the results changed after excluding these cases, we repeated the disproportionality analysis for the remaining 31 cases (Table 3). While the ROR (95% IC) values were lower compared to those of the first analysis, the results from the repeated disproportionality analysis still displayed a high disproportionality regarding gliptin-BP association for both the group analysis and the analysis by individual active ingredients separately. The order by active ingredient and gender prevailed.

**Table 3:** Sensitivity analysis for the association between DPP4i exposure and bullous pemphigoid reporting as measured by the disproportionality analysis based on FEDRA database

Active ingredient	Cases/non-cases	ROR (95% CI)	X^2^
*all IDPP4, total*	*31/1967*	*38.2 (24.9*–*58.4)*	*737.4*
Females	17/919	39.0 (22.2–68.2)	424.00
Males	14/924	19.2 (10.6–34.7)	174.1
*Vildagliptin, total*	*18/581*	*63.70 (38.0*–*106.7)*	*834.3*
Females	11/247	92.97 (47.5–181.0)	700.6
Males	7/278	31.59 (14.2–70.1)	152.87
*Linagliptin, total*	*8/302*	*42.74 (20.4*–*89.3)*	*251.4*
Females	*5/150*	77.8 (30.0–201.6)	258.9
Males	*3/148*	21.5 (6.6–70.0)	36.65
*Sitagliptin, total*	*5/979*	*9.31 (3.7*–*22.9)*	*27.5*
Females	*1/491*	4.41 (0.6–32.0)	0.3
Males	*4/479*	11.45 (4.1–31.8)	26.0
*Saxagliptin, total*	*0/1*	N. C.	N. C.
Females	0/0	N. C.	N. C.
Males	0/1	N. C.	N. C.

N. C.: not calculable

### Pooled analysis

**Fig. 1:**
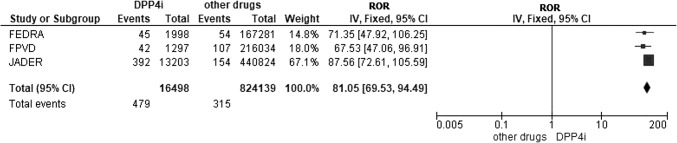
Forest plot off comparison DPP4i versus other drugs (outcome: bullous pemphigoid). Pooled ROR of data from three pharmacovigilance databases: FPVD, JADER and FEDRA

By combining the results of our study with those of the case/non-case studies conducted over the FPVD (France) and JADER (Japan) databases [5, 6], we obtained a pooled ROR value (95% CI) equal to 80.1 (69.5–94.5) for the association of gliptins and BP (Fig. 1). The analysis by active ingredients yielded the following ROR values: vildagliptin, 118.5 (101.7–138.0); linagliptin, 32.7 (24.6–43.4); saxagliptin, 17.4 (7.7–39.4), and sitagliptin, 12.9 (10.4–16.0).

### BP and metformin

**Fig. 2:**
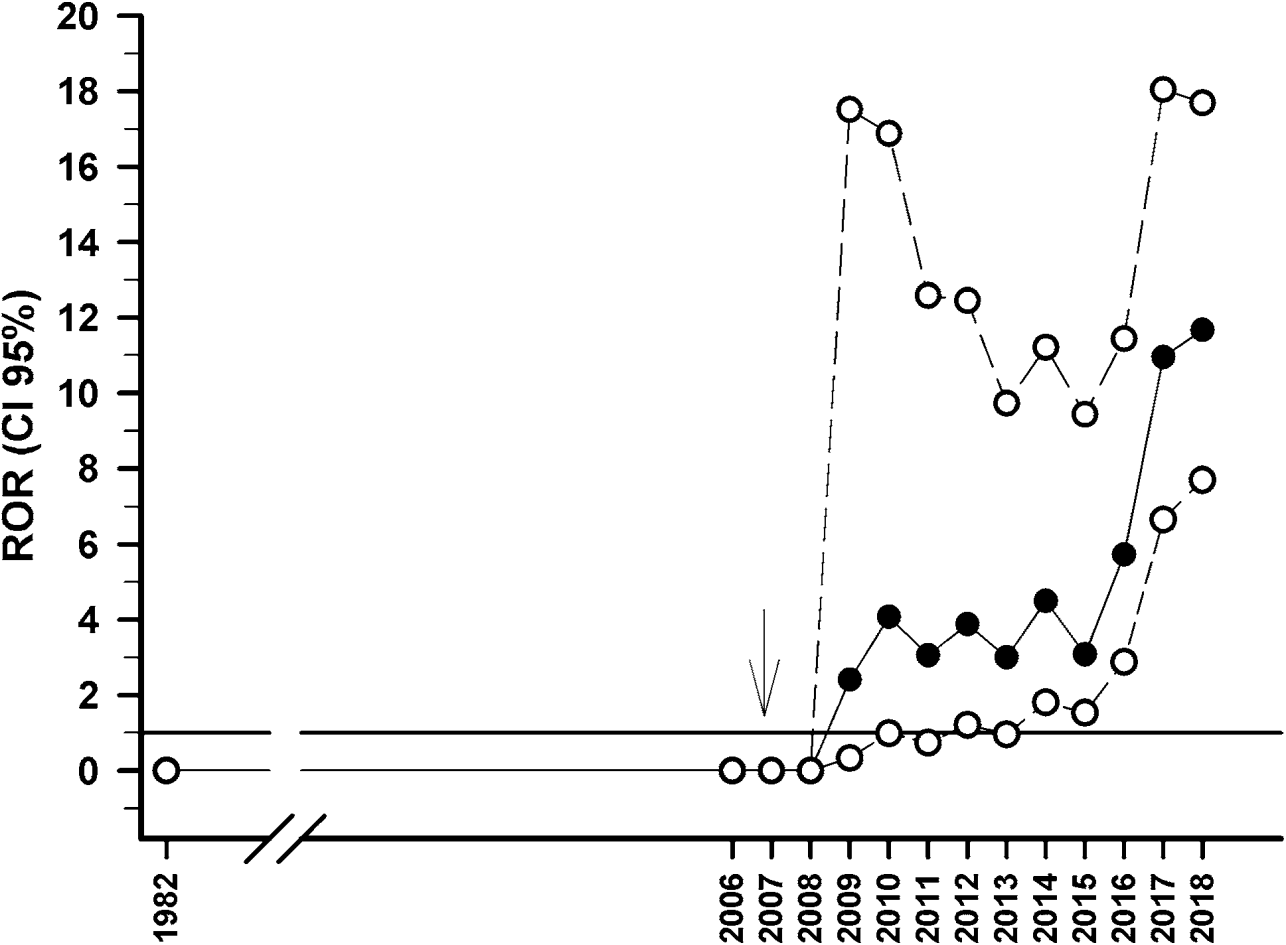
ROR (95% CI) value evolution for the BP-metformin association in FEDRA database. (filled circle: ROR central value; open circle: 95% confidence intervals; ↓: year of the first gliptin commercialisation approval)

In Spain, metformin was commercialised in 1982. FEDRA contained a total of 29 cases in which an association between BP and metformin was reported. In 21 of these, metformin was the suspected drug, whilst in the remaining 8 this medication was the concomitant drug. Figure 2 displays the annual evolution of ROR values for this association and the date on which the first gliptin commercialisation was approved.

In 2019, the ROR (95% CI) value for the BP-metformin association was 12.1 (7.9–18.5). The first notification including this association was submitted in 2009. In 26 (90%) of the cases of BP-metformin association, there was a gliptin as a co-administered medication as well. In the remainder 3 cases, notifications included the following medications: empagliflozin (1 case); ramipril, manidipine, and torasemide (1 case); and furosemide, hydroxyzine, insulin, paclitaxel, aceclofenac, hydrosmine, omeprazole, pertuzumab, racecadotril, and trastuzumab (1 case). In the case in which there was co-administration of metformin and empagliflozin, the patient was a woman who presented a BP outbreak after having been previously diagnosed with vildagliptin-associated BP (this case had been already included in our study). In the remaining 2 cases, the patients were on drugs whose association with BP risk has been well established, such as Ramipril [17], furosemide, and omeprazole [4].

## Discussion

### Overall results on BP-gliptin association

Herein, we report the results from the case/non-case analysis of the association between IDPP4 and BP. These results showed the disproportionality found in the Spanish database FEDRA, which was consistent with those reported in earlier studies conducted across several pharmacovigilance databases, showing an association between BP and a number of dipeptidyl peptidase-4 inhibitors [5, 6]. Furthermore, our findings are consistent with studies conducted in other countries [8, 18–21], which also demonstrated an association between these drugs and BP for both the entire pharmacological group and the individual drugs separately. The disproportionality extent on the basis of active ingredients coincides with that observed in the aforementioned studies as well, with this extent being the largest for vildagliptin, followed by linagliptin and sitagliptin. The cases of suspected bullous pemphigoid or pemphigus notified to the Spanish pharmacovigilance system (SEFV-H) increased considerably since 2008. This date roughly coincides with the start of gliptin use in Spain. In fact, gliptins were the drugs reporting the largest case number [22]. The global increase in DMT2 prevalence will rise the prescription of antidiabetic drugs, either as a monotherapy medication or co-administered with newly approved medications, as stated in 2019’s ADA recommendations [23]. Consequently, it is reasonable to expect the spontaneous reporting of BP cases to increase in the coming years.

### Patients’ demographic characteristics

In our study, most BP patients were over 70 years (median 75.5 and 79 year for females and males, respectively), which is in line with earlier studies. There was a striking case of a girl aged only 6.5 years. We did not find any differences regarding gender in our study.

### Potentially alternative causes/sensitivity analysis

We took into consideration other medications as potential BP causes, however the sensitivity analysis did not find any potentially alternative pharmacological causes to explain the occurrence of BP.

### Metformin-BP association

Metformin might be involved in the appearance of BP. While we found disproportionality in the association of metformin and BP, there was enough evidence to conclude that metformin’s role was casual rather than causal. Although metformin was approved for clinical use in Spain in 1982, reports of BP involving this drug were not submitted until 2009, a subsequent date to the commercialisation of the first gliptin. While in 29 cases metformin was involved in the occurrence of BP, we found that in these cases there were other drugs that could more convincingly explain the ADR. In 27 of these 29 cases, a gliptin was involved. It is worth mentioning that most patients treated with gliptins are expected to be concomitantly treated with metformin, since this is a recommendation in diabetes management guidelines (ADA, 2019) [23]. Although in our gliptin group metformin was involved in only 46.7% of the cases, it is reasonable to assume that more patients had metformin prescribed as a co-administered medication. On this matter, it should be noted that in many occasions only the suspected medication is reported to the pharmacovigilance systems. Therefore, we conclude that metformin was a misleading factor in our study.

#### Latency

Latency periods showed some differences respect to previous studies. Pasmatzi [24] reported that a latency of 2 months; Attaway [7] 6 months; and Bene [5] 1 month. In our study, latency was reached 7 months for IDPP4 inhibitors as a whole. Additionally, we found that the latency period was different for each IDPP4 inhibitor, the shortest one being for linagliptin (3.5 months), and the longest for sitagliptin (12 months).

Some delayed reactions begin weeks after continuous treatment. These reactions may be caused by several different mechanisms, but they are not IgE mediated [25].

#### Pooled analysis

To our knowledge, this is the first study collecting data from three different national spontaneous reporting databases (i.e. French, Japanese, and Spanish) under the MedDRA preferred term (PT) “bullous pemphigoid” associated with gliptins. Likewise, ours is the first study weighting the estimators for each national database aiming to obtain a pooled estimator. At any rate, our pooled estimator did not include the estimators based on studies conducted in other countries, such as Finland [19] or Korea [21], because such investigations were not case/non-case studies based on a pharmacovigilance database. Our pooled estimator for report risk is consistent with the data from the three databases regarding the entire pharmacological class and also each one of the researched gliptins. When we cross-referenced the information from the three national databases, vildagliptin presented the highest association value. We did not find any ethnic differences (i.e. Caucasian vs. Asian), which suggests that BP is not related to a genetic alteration.

### Study limitations

In different Spanish regions [26, 27], as well as in other world regions [28, 29], ADRs may be under-reported. However, in 2018, Maciá [30] conducted a meta-analysis based on 15 studies published in Eudravigilance using a logarithm regression model, which found that the proportional reporting rate (PRR) and the relative risk estimator (RRE) presented a statistically significant co-relationship. Furthermore, this author reported that the variables PRR and RRE in the national Spanish pharmacovigilance database (FEDRA) moved together in the same direction. Thus we believe that, despite we did not estimate the PRR and we were not able to use the model utilised by Maciá [30], the data in our study may move in the same direction as well. Consequently, we should not overlook the possibility that the findings based on pharmacovigilance databases, which seem to be corroborated by the results from the present study, may be able to elucidate some factors which could reduce BP risk associated with the use of oral hypoglycaemic agents.

## Conclusion

The combined analysis of studies from French, Japanese, and Spanish pharmacovigilance databases suggests an association between iDDP-BP, where most affected patients were over 70 years old. No gender differences were found. The association would be stronger in the case of vidagliptin, followed by linagliptin, and there was enough evidence to conclude that mefformin´s role was casual rather than causal regarding BP. Future studies, particularly clinical trials, are required in order to establish real incidence and confirm cause-effect relationships.
